# Detailed investigations of proximal tubular function in Imerslund-Gräsbeck syndrome

**DOI:** 10.1186/1471-2350-14-111

**Published:** 2013-10-24

**Authors:** Tina Storm, Christina Zeitz, Olivier Cases, Sabine Amsellem, Pierre J Verroust, Mette Madsen, Jean-François Benoist, Sandrine Passemard, Sophie Lebon, Iben Møller Jønsson, Francesco Emma, Heidi Koldsø, Jens Michael Hertz, Rikke Nielsen, Erik I Christensen, Renata Kozyraki

**Affiliations:** 1 Department of Biomedicine, Aarhus University, Aarhus, Denmark; 2 INSERM UMR S968, Institut de la Vision, Paris, 75012, France; 3 UPMC Univ Paris 06, UMR_S 968, Institute de la Vision, Paris, F-75012, France; 4 CNRS, UMR_7210, Paris, F-75012, France; 5 Service de Biochimie B, Hôpital Saint-Antoine, Paris, France; 6 Laboratoire de Biochimie, Hôpital Robert Debré, Paris, France; 7 Service de Génétique, Hôpital Robert Debré, Paris, France; 8 Inserm U676, Hôpital Robert Debré, Paris, France; 9 Department of Pediatrics, Aarhus University Hospital, Aarhus, Denmark; 10 Department of Nephrology and Urology, Division of Nephrology and Dialysis, Ospedale Bambino Gesù, IRCCS, Rome, Italy; 11 Centre for Insoluble Protein Structures (inSPIN) and Interdisciplinary Nanoscience Center (iNANO), Department of Chemistry, Aarhus University, Aarhus, Denmark; 12 Department of Clinical Genetics, Odense University Hospital, Odense, Denmark

**Keywords:** Imerslund-Gräsbeck syndrome, Cubilin, Amnionless, Proximal tubules, Tubular proteinuria

## Abstract

**Background:**

Imerslund-Gräsbeck Syndrome (IGS) is a rare genetic disorder characterised by juvenile megaloblastic anaemia. IGS is caused by mutations in either of the genes encoding the intestinal intrinsic factor-vitamin B_12_ receptor complex, cubam. The cubam receptor proteins cubilin and amnionless are both expressed in the small intestine as well as the proximal tubules of the kidney and exhibit an interdependent relationship for post-translational processing and trafficking. In the proximal tubules cubilin is involved in the reabsorption of several filtered plasma proteins including vitamin carriers and lipoproteins. Consistent with this, low-molecular-weight proteinuria has been observed in most patients with IGS. The aim of this study was to characterise novel disease-causing mutations and correlate novel and previously reported mutations with the presence of low-molecular-weight proteinuria.

**Methods:**

Genetic screening was performed by direct sequencing of the *CUBN* and *AMN* genes and novel identified mutations were characterised by *in silico* and/or *in vitro* investigations. Urinary protein excretion was analysed by immunoblotting and high-resolution gel electrophoresis of collected urines from patients and healthy controls to determine renal phenotype.

**Results:**

Genetic characterisation of nine IGS patients identified two novel *AMN* frameshift mutations alongside a frequently reported *AMN* splice site mutation and two *CUBN* missense mutations; one novel and one previously reported in Finnish patients. The novel *AMN* mutations were predicted to result in functionally null *AMN* alleles with no cell-surface expression of cubilin. Also, the novel *CUBN* missense mutation was predicted to affect structural integrity of the IF-B_12_ binding site of cubilin and hereby most likely cubilin cell-surface expression. Analysis of urinary protein excretion in the patients and 20 healthy controls revealed increased urinary excretion of cubilin ligands including apolipoprotein A-I, transferrin, vitamin D-binding protein, and albumin. This was, however, only observed in patients where plasma membrane expression of cubilin was predicted to be perturbed.

**Conclusions:**

In the present study, mutational characterisation of nine IGS patients coupled with analyses of urinary protein excretion provide additional evidence for a correlation between mutation type and presence of the characteristic low-molecular-weight proteinuria.

## Background

Imerslund-Gräsbeck Syndrome or Megaloblastic Anaemia 1 (IGS or MGA1, OMIM #261100) is a rare, autosomal recessive disorder characterised by selective intestinal vitamin B_12_ malabsorption [1,2]. Most common clinical features of the syndrome include megaloblastic anaemia, failure to thrive, recurrent infections and selective low-molecular-weight proteinuria [3]. IGS is a heterogenic disorder caused by mutations in *CUBN* or *AMN*[4,5]. It was originally described simultaneously in Norway and Finland in 1960 [1,2] and since then, several hundred cases have been reported worldwide [6]. A number of these cases, however, may very likely represent misdiagnosed patients suffering from mutations of the gastric intrinsic factor gene (*GIF*) [7]. Both disorders, IGS and hereditary *GIF* dysfunction, result in vitamin B_12_ deficiency and are clinically very difficult to distinguish [6]. Especially, has the diagnosis of a group of IGS patients presenting without proteinuria proved challenging to tell apart from patients with juvenile dysfunction of *GIF*. Until recently, these patients were clinically distinguished on the basis of the so-called Schillings test [8] revealing any deficiency in functional intrinsic factor. However, the test is no longer available and the two groups of patients may today only be correctly diagnosed through genetic analyses of the genes involved [6].

**Figure 1:**
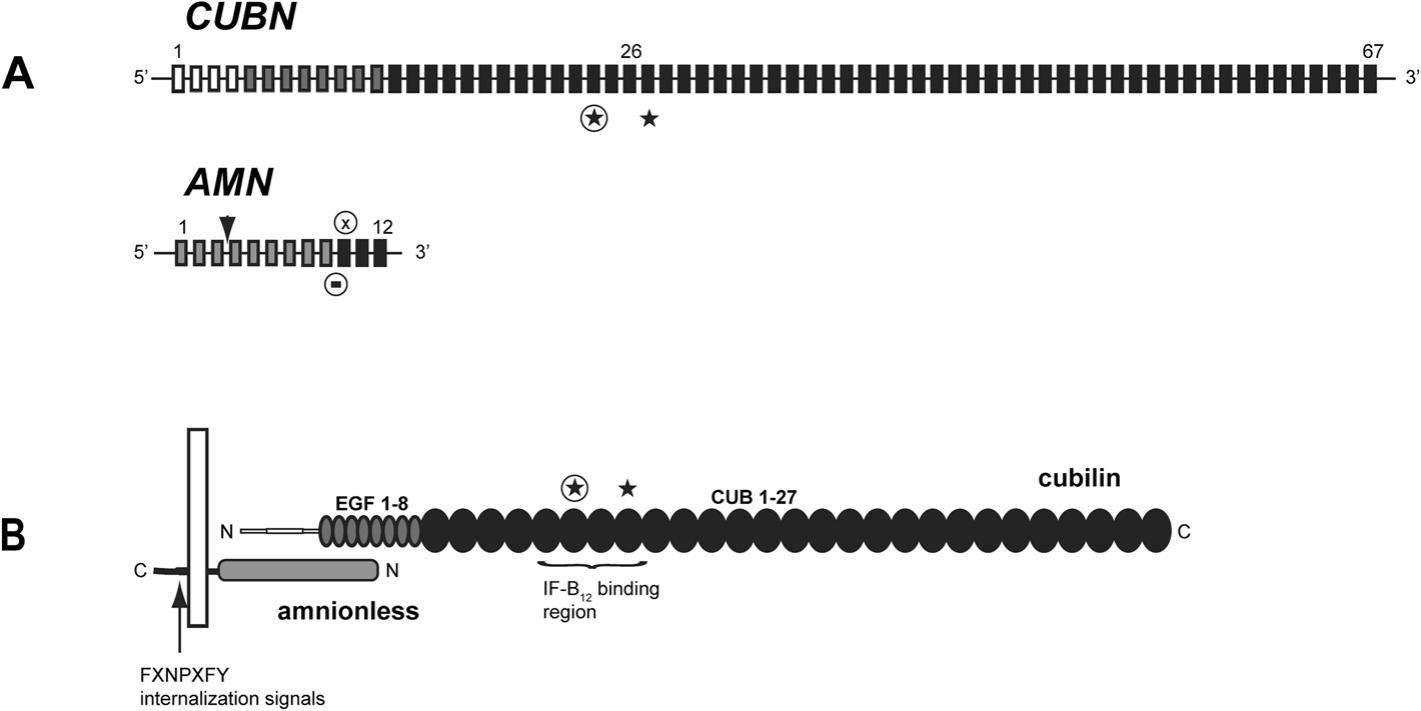
**Schematic presentation of the *CUBN/AMN *genes and translation products. A**: Schematic presentation of the genomic structure of the *CUBN* and *AMN* genes. The previously described and novel *CUBN* missense mutations are indicated by a star and an enclosed star respectively. The novel *AMN* mutation deletion and insertion/deletion mutations are indicated by an enclosed bar and X respectively and the previously described AMN founder mutation by an arrowhead. Exons are color-coded in grey scale to match the corresponding regions in the translated proteins for easy gene-to-function coupling. **B**: Domain organisation in the two translation products cubilin and amnionless. EGF and CUB domains of cubilin are shown as grey and black eclipses. Intrinsic factor-vitamin B_12_ binding region with identified missense mutations are furthermore highlighted. Amnionless is depicted with c-terminal membrane association and extracellular cubilin interaction.

*CUBN* is located on chromosome 10 and encodes cubilin (Figure 1B), also known as the intrinsic factor-vitamin B_12_ (IF-B_12_) receptor [9,10] whereas *AMN* maps to chromosome 14 and encodes amnionless (Figure 1B), a 50 kDa, type 1 transmembrane protein [11]. Cubilin is a 460 kDa membrane-associated receptor protein comprising multiple ligand-binding CUB (*C*omplement subcomponents C1r/s, *U*egf, and *B*mp 1) domains [12,13]. Together, cubilin and amnionless constitute the receptor complex cubam, responsible for intestinal IF-B_12_ uptake [14]. The IF-B_12_ binding site has been located to cubilin CUB domains 5–8 [15] and recently the structural basis for the interaction was established [16].

We and others have demonstrated a highly interdependent relationship of cubilin and amnionless for correct processing and apical trafficking to the plasma membrane [14,17-24]. So far, no transmembrane segment or endocytic signals have been identified in cubilin [12]. Amnionless however, harbours signals for receptor-mediated endocytosis through clathrin-coated vesicles [14] and may mediate internalization of the intestinal IF-B_12_ receptor complex by engaging the clathrin-associated sorting proteins disabled-2 (Dab2) and autosomal recessive hypercholesterolemia (ARH) [25]. Cubilin and amnionless are both highly expressed in the small intestine as well as the proximal tubules of the kidney. In the latter, they functionally interact with the multi-specific endocytic receptor megalin allowing the reabsorption of a panel of filtered plasma proteins [26]. Cubilin is particularly important for the normal tubular reabsorption of albumin [27,28], vitamin D-binding protein (VDBP) [29,30], apolipoprotein A-I (apo A-I) [31], and transferrin [32] but only the binding of albumin has been mapped to cubilin so far [33].

Several *CUBN* and *AMN* mutations have been reported [24,34,35] since IGS was first reported in the 1960’s. Most reported mutations of the *AMN* gene most likely represents functional null mutations. One splice-site mutation in particular has been reported a number of times in the Mediterranean region [5,36-38]. This mutation changes the acceptor splice site of *AMN* intron 3 (c.208-2A>G) and causes skipping of exon 4, resulting in a frameshift and premature stop codon in exon 6 [5]. Based on the established interdependent relationship of cubilin and amnionless the *AMN* intron 3 mutation most likely affects processing and trafficking of cubilin although this remains to be demonstrated.

Investigations of a canine IGS model have furthermore provided valuable insight into the role of amnionless in this syndrome [20]. The IGS dogs suffer from functional null mutations of the canine homologue to human *AMN*. Immunohistochemical investigations of renal tissue from these dogs showed that cubilin was not expressed at the surface but retained in intracellular vesicles [20]. This clearly illustrates a vital role of amnionless in normal cubilin trafficking and membrane expression and furthermore links mutations of *AMN* with intestinal malabsorption of IF-B_12_.

The most prevalent mutation found in Finnish patients (FM1) is a *CUBN* missense mutation changing the highly conserved proline 1297 of cubilin CUB domain 8 to a leucine [4]. This substitution results in decreased affinity of the IF-B_12_ complex [39] hereby linking the underlying genetic mutation with the intestinal malabsorption of IF-B_12_ in these patients. In addition, a number of sporadic *CUBN* null and missense mutations have also been identified [23,34,35]. However, the functional implications have only been reported in two patients. A single cytosine for guanine exchange in *CUBN* intron 23 (c.3330-439C>G, originally described as IVS-intra CUB6 C>G, FM2) was found to trigger complex splicing, resulting in premature truncation of the receptor protein [4]. In addition, we recently investigated another sporadic *CUBN* mutation in an Italian family [23]. The mutation changed the highly conserved donor splice site of exon 23 most likely resulting in aberrant splicing and functionally null *CUBN* alleles. Accordingly, no cubilin was detected in renal tissue from this patient [23].

Recently, we established that the molecular background for the low-molecular-weight proteinuria observed in these patients is due to proximal tubular dysfunction of cubilin [23]. Immunohistochemical investigations of renal tissue from a cubilin-deficient patient revealed an abnormal distribution of the receptor partner amnionless as well as reduced uptake of the selective cubilin ligand apo A-I. Furthermore, analyses of the urinary protein excretion in this patient revealed increased urinary excretion of the cubilin ligands transferrin, apo A-I, VDBP, and albumin as previously reported [28,30-32,40]. Consistent with observations from the dogs suffering from mutations of the canine *AMN* homologue, low-molecular-weight proteinuria has also been reported in IGS patients with *AMN* mutations [24,36-38].

Interestingly, the characteristic low-molecular-weight proteinuria has not been consistently observed in IGS patients [40]. Correlation between the specific disease-causing mutations and the low-molecular-weight proteinuria has not been established so far but accumulating evidence indicates that functional null mutations of both *CUBN* and *AMN* may result in low-molecular-weight proteinuria contrasting observations from patients with the FM1 missense mutation [24,36-38,40].

In the present study, genetic screening of nine IGS patients identified two previously described disease-causing mutations as well as three novel mutations, including two *AMN* null mutations and one *CUBN* missense mutation. Functional investigations of the novel mutations predicted them to result in dysfunctional membrane expression of cubilin. Low-molecular-weight proteinuria was only identified in patients where cubilin was predicted to be absent from the cell surface, thus, providing additional evidence for a correlation between the nature of the genetic mutations and the characteristic urinary protein excretion observed in most of these patients.

## Methods

### Patients

Informed consent for genetic testing was obtained from all the families included in this study. This study was approved by the Central Denmark Region Committee on Biomedical Research Ethics and conducted in accordance with the Declaration of Helsinki. Genomic DNA was isolated from peripheral blood using standard procedures for direct genomic sequencing of *CUBN* and *AMN* genes. Diagnosis of selective intestinal vitamin B_12_ malabsorption (IGS or MGA1) was based on previously established criteria [6]. For detailed patient information see Additional file 1. Family information is given in Table 1.

**Table 1:** Family information

**Family**	**Consanguineous parents**	**Ethnic origin**	**Number of affected children**
***AMN***			
**Family 1**			
*c.208-2A>G*^*A*^*/c.208-2A>G*^*A*^	*yes*	*Turkish*	*1*
*(p.Leu70Alafs)*			
**Family 2**			
*c.208-2A>G*^*A*^*/c.208-2A>G*^*A*^	*yes*	*Tunesian*	*2*
*(p.Leu70Alafs)*			
**Family 3**			
*c.1006 + 11_1008del/*			
	*yes*	*Moroccan*	*1*
*c.1006 + 11_1008del*			
*(p.Glu337Asnfs)*			
**Family 4**			
c.1041_1042delinsCTC/			
	*no*	*Italian*	*2*
*c.208-2A>G*^*A*^			
*(*p.Glu348Serfs/*p.Leu70Alafs)*			
***CUBN***			
**Family 5**			
*c.3335G>A/c.3335G>A*	*yes*	*Tunesian*	*2*
*(p.Gly1112Glu)*			
**Family 6**			
*c.3890C>T/c.3890C>T*^*B*^	*no*	*Finnish*	*1*
*(p. Pro1297Leu)*			

Table 1 summarises the families included in the study with identified mutations, family background and number of affected children.

^A^Previously described as *c.208-2A>G, skipping of exon 4, fs*[5]. ^B^Previously described by [4]. Paternal/maternal allele.

### Mutation analyses

Nucleotides are numbered according to GenBank accession numbers [NM_001081.3] (*CUBN*) and [NM_030943.3] (*AMN*) with +1 corresponding to the A of the ATG translation initiation codon and the initiation codon corresponding to codon 1. *CUBN* and *AMN* exons, with flanking intronic regions, were amplified using standard PCR procedures with sequence specific primers (primer sequences are available upon request) and a polymerase (HOT FIREPol^®^ DNA polymerase; Solis Biodyne, Estonia). *AMN* exons were amplified in 7 fragments with addition of the PCR additive S-Solution for amplification of GC-rich templates. Amplified products were enzymatically purified (ExoSAP-IT; USB Corporation, Cleveland, Ohio, USA) and used as template in sequencing reactions (Big Dye^®^ Terminator v1.1 Cycle Sequencing Kit; Applied Biosystems, Naerum, Denmark). Sequencing products were purified using pre-soaked Sephadex G-50 (GE Healthcare Orsay, France) 96-well multiscreen filter plates (Millipore, Molsheim, France). Purified products were analysed on an automated 48-capillary sequencer (ABI 3730 Genetic analyser; Applied Biosystems, Courtaboeuf, France) and the results interpreted using the SeqScape^®^ software (Applied Biosystems). Novel sequence variants were compared to commercially available control alleles (Human random control panels; Health Protection Agency Culture Collections, Salisbury, United Kingdom) to exclude commonly occurring polymorphisms. *In silico* splicing prediction analyses was performed using the NNSPLICE server (0.9 version) (http://fruitfly.org/seq_tools/splice.html). No additional patient material was available for analyses of *AMN* splicing.

### Established constructs

The genomic fragments comprising *AMN* exon 8 to exon 12 (c.782-1327) were amplified through PCR from a healthy control subject and the index patient of family 3 (homozygous for c.1006 + 11_1008del) using the following *AMN* specific primers; 5′-CCCTCCCGCTAGCATGGCCGTTGTGTTGCTGACCCA-3′ containing the NheI restriction site and an in frame ATG start codon and primer 5′-ATTCCCCTCGAGTCATGACGAAGTAACTGTGGCTGGT-3′ containing the Xho I restriction site and an in frame TGA stop codon. Amplification products were cloned into expression vectors (pcDNA™ 3.1(+); Invitrogen, Taastrup, Denmark) using NheI-HF™ and Xho I restriction enzymes (New England Biolabs, Medinova Scientific A/S, Glostrup, Denmark) according to manufacturer’s guidelines (wt gAMN8-12 and del gAMN8-12) and subsequently transformed into competent cells (One Shot^®^ TOP10; Invitrogen) for propagation. CHO-K1 cells were transiently transfected with either wt gAMN8-12 or del gAMN8-12 plasmids in duplicates and total RNA was isolated 48 hours after transfection (RNeasy Mini Kit; Qiagen, Ballerup, Denmark). Splicing was analysed by RT-PCR (OneStep RT-PCR kit; Qiagen) using primers 5′-CACCTTCCTGGGTCTGCCTCAGTACC-3′ and 5′-GGCGCCACCAGCAGGACCAGCA-3′ according to manufacturer’s protocol. cDNA amplification products were cloned into vectors (pCR^®^II-TOPO^®^ using TOPO TA cloning technique, Invitrogen) according to manufacturers protocol for subsequent sequencing.

The c.3335G>A (p.Gly1112Glu) identified in family 5, was introduced into a previously described fragment of human cubilin, encoding CUB domains 5–8 [39], through site-directed, ligase-independent mutagenesis (SLIM)[41] using the following primers (Ft: 5′-CAGAGATGAAGGCTATGAAAAATCACCATTGCTGGG-3′; Rt: 5′-TCATAGCCTTCATCTCTGATTTCCAGAAAATCTGTA-3′; Fs: 5′-AAAATCACCATTGCTGGG-3′; Rs: 5′-ATTTCCAGAAAATCTGTA-3′). Whole plasmid amplification was carried out in one reaction using a polymerase (Herculase II fusion polymerase; AH Diagnostics a/s, Aarhus, Denmark) with subsequent digestion of template strands and rehybridisation as previously described [41]. Plasmids were subsequently transformed into competent cells (One Shot^®^ TOP10, Invitrogen) for propagation.

### Cell propagation and transfection

CHO-K1 cells were grown in HyQ-CCM5 (HyClone, Logan, UT, USA) serum free growth medium containing 100 units/ml penicillin and 100 μg/ml streptomycin and transiently transfected with plasmids using the Lipofectamine™ 2000 (Invitrogen) according to manufacturer’s protocol.

### Preparation of cell lysates and conditioned growth medium

Growth medium was collected from propagated cells prior to lysis and centrifuged at 2,000 g for 2 min. Supernatant was transferred to a new tube, sample buffer added and concentrated for 50 min at 95°C. Cells were washed twice with PBS buffer pH 7.4 and subsequently lysed using a Triton X-100 (Merck, Denmark, Industrial Chemicals & Pigments, Hellerup, Denmark) and EDTA-free protease inhibitor (Complete; Roche, Hvidovre, Denmark) containing buffer. Cell lysates were centrifuged at 4,000 g for 5 min and supernatants collected. Growth medium and cell lysates were subsequently analysed using SDS-PAGE and immunoblotting.

### Urine analyses

Urine samples (24-hour urine or spot urine) obtained from the patients were frozen at -80°C after collection. Urinary protein excretion was normalised using urinary creatinine concentrations and compared with urines collected from 20 young, healthy subjects (aged 3–7 years). The urinary excretion of a certain ligand was defined as increased when all of the controls despite variability had excretion levels below the excretion levels observed in patients. Urinary protein excretion was evaluated through immunoblotting or using the Sebia High-Resolution Gel Electrophoresis System (Sebia, Evry, France) according to the manufacturer’s instructions. Urine samples were applied on high resolution gels for urine analysis (Hydragel 5 proteinuria; Sebia) and processed using the Hydrasys 2 instrument (Sebia).

### Immunoblotting

Proteins were separated by SDS-PAGE and transferred to an ImmobilonTM–FL PVDF transfer membrane (Millipore, Copenhagen, Denmark) using the iBlot™ Dry Blotting System (Invitrogen). Membranes were subsequently blocked and incubated with primary and fluorophore-coupled secondary antibodies according to manufacturer’s instructions (LI-COR Biosciences, Cambridge, United Kingdom). Proteins were detected using the Odyssey™ infrared imager (LI-COR Biosciences).

### Antibodies

(*Primary)* rabbit anti vitamin D-binding protein (Dako, Glostrup, Denmark), rabbit anti transferrin (Dako), rabbit anti apo A-I (Dako), rabbit anti albumin (Dako), rabbit anti α_1_-M (Dako), rabbit anti retinol-binding protein (RBP) (Dako), rabbit anti human cubilin (kindly provided by Søren K. Moestrup, Institute of Biomedicine, Aarhus University, Denmark) (*Secondary*) goat anti-rabbit IRDye^®^-800 CW (LI-COR Biosciences, Lincoln, Nebraska USA).

## Results

### Mutation analyses

Direct sequencing of the *CUBN* and *AMN* genes in six affected families (1–6) (Table 1) revealed two novel mutations of the *AMN* gene (Family 3 and 4) and one novel mutation of the *CUBN* gene (Family 5). In families 1 and 2 a homozygous mutation of the *AMN* intron 3 acceptor splice site was identified (c.208-2A>G). This mutation was originally reported in families of Tunisian Jewish and Turkish origin as c.208-2A>G, skipping of exon 4; fs [5,34] and causes complete skipping of exon 4 resulting in a frameshift and a premature stop codon (Table 2).

**Table 2:** Functional characterisation of identified IGS causing mutations

**Family**	**Gene**	**Genomic region**	**Protein prediction**	**Mutation type**	**Protein region**	**Low–molecular-weight Proteinuria**	**Predicted cubilin cell-surface expression**
	***AMN***						
1 and 2	*c.208-2A>G*^*A*^*/c.208-2A>G*	Intron 3	p.Leu70Alafs	Frameshift	Extracellular domain	Yes^*^	No^A^
3	*c.1006 + 11_1008del/*	Intron 9	p.Glu337Asnfs	Frameshift	Extracellular domain	Yes^*^	No^**†** and ♦^
	*c.1006 + 11_1008del*						
4	*c.1041_1042delinsCTC*/	Exon 10/	p.Glu348Serfs/	Frameshift/	Extracellular domain/	Yes^*^	No^♦^
	*c.208-2A>G*^*A*^	intron3	p.Leu70Alafs	Frameshift	Extracellular domain		
	***CUBN***						
5	*c.3335G>A/c.3335G>A*	Exon 24	p.Gly1112Glu	Missense	CUB 6	Yes^*^	No^**†** and ♦^
6	*c.3890C>T/c.3890C>T*^*B*^	Exon 27	p.Pro1297Leu	Missense	CUB 8	(no)^*^	Yes^C^

Table 2 summarises functional characterisation and protein predictions of the identified mutations including mutation classification, observations of low-molecular-weight proteinuria, prediction of cubilin membrane expression and receptor function. *Based on analyses of urinary excretion of selective cubilin and megalin ligands in patient urines using immunoblotting or high-resolution gel electrophoresis, see Table 3. ^†^Based on *in vitro* functional analyses. ^♦^Based on *in silico* protein predictions.^A^Previously described as *c.208-2A>G, skipping of exon 4, fs*[5]. ^B^Previously described by [4]. ^C^Functional analyses previously described by [39].

**Figure 2:**
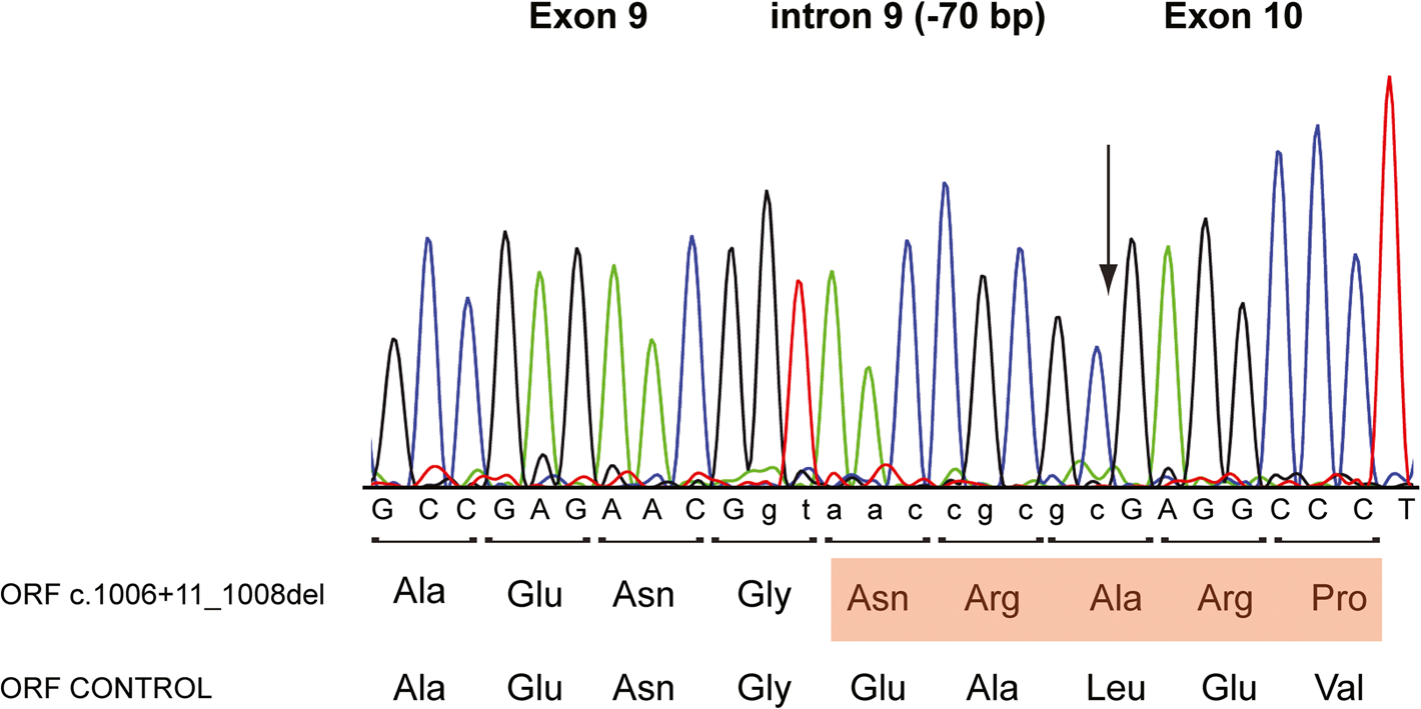
**Sequencing analysis of (c.1006 + 11_1008del) gAMN8-12 minigene mRNA transcript.** A selected region of the trace data, obtained from RT-PCR analyses of purified mRNA from transiently transfected CHO-K1 cells with the established (c.1006 + 11_1008del) gAMN8-12 minigene construct, showed incorporation of 10 base pairs of *AMN* intron 9 in the spliced mRNA product. Arrow indicates point of the 70 bp deletion. Coding and intron sequence is represented by upper and lower case letters, respectively. The open reading frame (ORF) for normally spliced *AMN* mRNA in this region is shown below with the resulting altered reading frame for c.1006 + 11_1008del highlighted in red.

In family 3, direct sequencing revealed a homozygous 70 bp deletion of *AMN* intron 9 (c.1006 + 11_1008del) with a resulting elimination of the *AMN* exon 10 splice acceptor site (Figure 1A) (Table 1). No alternative acceptor splice sites could be detected in *AMN* intron 9 or exon 10 using the NNSPLICE server (0.9 version). RT-PCR and cDNA sequencing of total RNA isolated from CHO-K1 cells expressing either genomic wt *AMN* exon 8–12 or *AMN* exon 8–12 (c.1006 + 11_1008del) showed abrogated splicing with retention of the remaining 10 bp of intron 9 in the mutant mRNA transcript (Figure 2). The deletion consequently causes a translational frameshift and a premature stop codon most likely resulting in nonsense mediated decay of the aberrant mRNA and thus functional *AMN* null alleles (Table 2). Consistent with this, both parents were heterozygous at this position and the 70 bp deletion was not detected in 158 control alleles.

Direct sequencing, of the *AMN* gene in family 4, revealed compound heterozygous mutations in the two affected children (Table 1). In both patients the c.208-2A>G mutation, described above, was observed on the maternal allele whereas a novel deletion-insertion of exon 10 (c.1041_1042delinsCTC) was identified on the paternal allele (Figure 1A). The insertion-deletion resulted in a translational frameshift and no alternative stop codon could be detected upstream of the *AMN* 3′UTR region (Table 2). Due to the aberrant and elongated *AMN* transcript product, the c.1041_1042delinsCTC may result in an unstable amnionless protein and thus in functional null alleles. The mutation was not seen in 166 control alleles.

**Figure 3:**
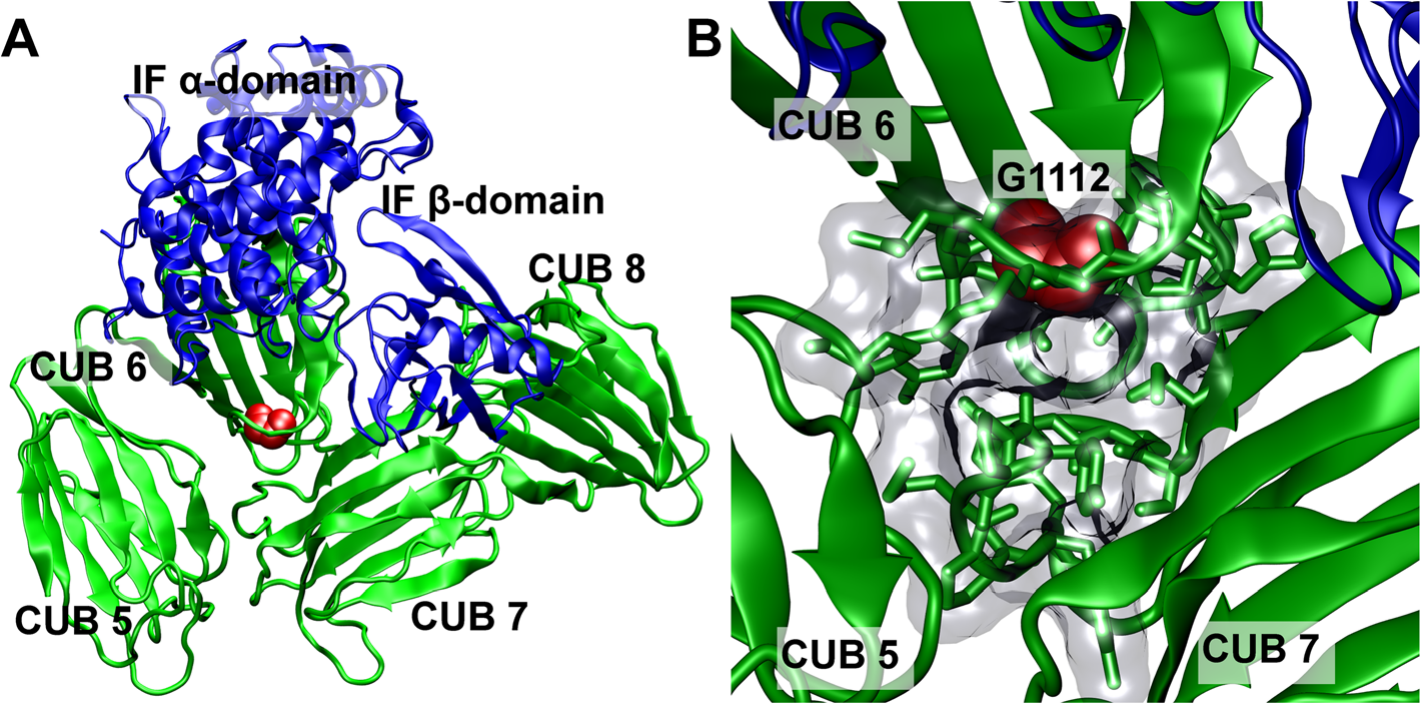
**G1112 is located in the tight interface between CUB 5, 6 and 7. A**: Overview of the structure of cubilin CUB domains 5–8 in complex with IF (PDB accession 3KQ4) [16]. The CUB domains are illustrated in green cartoons and IF domains in blue cartoons. The G1112 residue is located in the interface between CUB domains 5, 6 and 7 and shown as red spheres. **B**: Zoom-view of the interface between CUB domains 5, 6 and 7. Residues lining the interface are here indicated in green sticks. The space occupied by the interface residues is indicated by a transparent surface. This clearly shows that G1112 is centrally located within the tight interface and accordingly, that a substitution to a larger and polar residue could potentially cause severe sterical clash with surrounding residues as well as disturb inter-domain interactions. The figure was generated using Visual Molecular Dynamics [43].

In family 5, a novel missense mutation of *CUBN* exon 24 was identified (c.3335G>A, p.G1112E) (Figure 1A and B) (Table 1). *CUBN* exon 24 partly encodes CUB domain 6, part of the IF-B_12_ binding site in cubilin [15,16]. The mutation changes the fully conserved, small, non-polar residue glycine 1112 to a large, polar glutamate and was not detected in 350 control alleles. Glycine 1112 is located in loop 6 of CUB domain 6 near the interfaces of CUB domains 5, 6 and 7 [16] (Figure 3A and B). The interfaces are here dominated by Van der Waal’s interactions indicating that the introduction of a polar glutamate residue in this region could affect the structural integrity of the interacting CUB domains [16]. Consistent with this, introduction of the glutamate residue in this region was found to be probably damaging with a score of 1.0 by PolyPhen-2 v2.1 [42].

**Figure 4:**
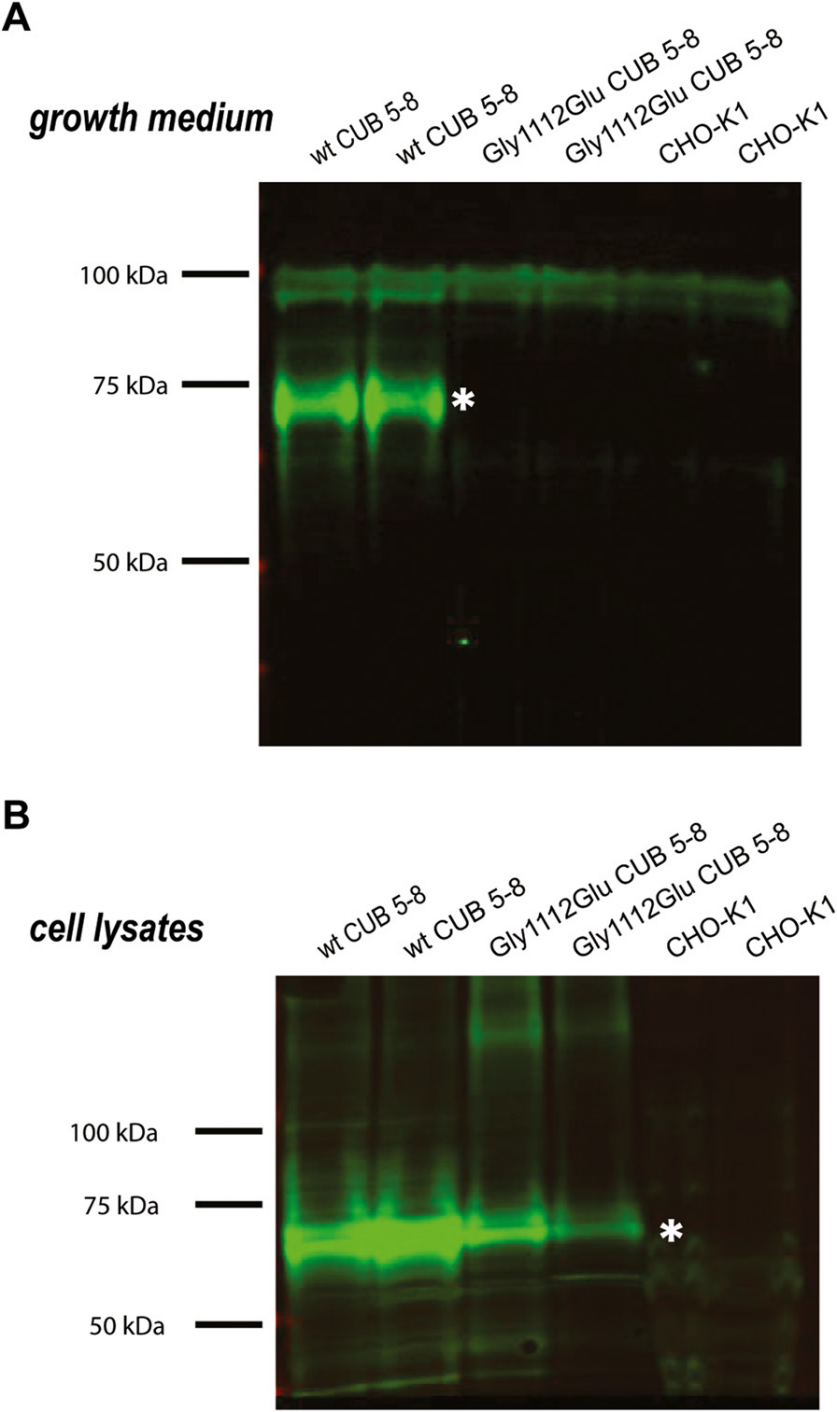
**Immunoblotting of conditioned growth medium (A) and cell lysates (B) from CHO-K1 cells transiently transfected with wt or Gly1112Glu CUB 5–8 cubilin.** Proteins were separated using SDS-PAGE (3–8%). and transferred to an Immobilon™ –FL PVDF transfer membrane using the iBlot™ Dry Blotting System. CUB 5–8 proteins were detected with a rabbit anti human cubilin antibody and visualised using LICOR IRDye λ 800 goat a-rabbit. Immunoreactive bands, consistent with the size of recombinant cubilin CUB 5–8, are indicated with white asterisks. No mutant CUB 5–8 protein was detected in the conditioned growth medium in contrast to the wt CUB 5–8 protein as previously described [39]. Both the wt and mutant protein were detected in cell lysates.

Cubilin CUB domains 5–8 [39] were expressed in two variants in CHO-K1 cells, a wild-type and a G1112E mutant form. Immunoblotting analyses of conditioned growth medium and cell lysates from CHO-K1 cells transiently transfected with wt or G1112E CUB 5-8 showed that the wt CUB 5-8 protein was secreted to the growth medium (Figure 4A) as previously reported [39]. However, the G1112E CUB 5-8 was only observed in cell lysates (Figure 4B) indicating that the mutant cubilin fragment is retained in intracellular compartments. This hereby suggests that the G1112E mutation identified in family 5 may have detrimental effects on the structural integrity of full length cubilin, possibly resulting in impaired processing and decreased or absent cell-surface expression of cubilin. Therefore, intestinal IF-B_12_-absorption in individuals of family 5 would probably be affected (Table 2).

Finally, direct sequencing revealed a homozygous missense mutation in *CUBN* exon 27 (c.3890C>T, p.P1297L) in the patient of family 6 (Figure 1B) (Table 1). This mutation has previously been reported in families of Finnish origin [4] and reduces IF-B_12_ complex recognition by cubilin (Table 2) [39].

### Urine analyses

Immunoblotting or high-resolution gel electrophoresis of urine collected from the patients of families 1–5 showed increased urinary excretion of the cubilin ligands; transferrin, apo A-I, albumin, VDBP, and α1-microglobulin (α_1_-M), recently described as a novel ligand of cubilin (Table 3) [23]. In line with a previous report of urinary protein excretion in FM1 patients [40], analyses of urinary protein excretion in the index patient of family 6 did not show clearly increased urinary excretion of albumin, transferrin or VDBP (Table 3). Also, no increased urinary excretion of the megalin ligand RBP was observed in the patient urines consistent with an isolated tubular cubilin dysfunction.

**Table 3:** Urinary protein excretion

**Mutation**	**Albumin**	**Transferrin**	**VDBP**	**Apo A-I**	**α**_**1**_**-M**	**RBP**
***AMN***						
*c.208-2A>G/c.208-2A>G*^*A*^	*x*	*x*	*x*	*No + x*	x	*No*
(p.Leu70Alafs)						
*c.1006 + 11_1008del/c.1006 + 11_1008del*	*x*	*x*	*x*	x	x	*No*
*(p.Glu337Asnfs)*						
c.1041_1042delinsCTC/*c.208-2A>G*^*A*^	*x*	*x*	*x*	x	*x*	*No*
*(*p.Glu348Serfs/p.Leu70Alafs)						
***CUBN***						
*c.3335G>A/c.3335G>A*	*x*	*x*	*x*	x	x	*No*
*(p.Gly1112Glu)*						
*c.3890C>T/c.3890C>T*^*B*^	*(x)*	*(x)*	*(x)*	*(x)*	*(x)*	*No*
*(p.Pro1297Leu)*						

Table 3 summarises the urinary protein excretion in the six families according to identified mutations. Urines were analysed by immunoblotting or high-resolution gel electrophoresis for urinary protein excretion of the cubilin ligands albumin, transferrin, VDBP, Apo A-I, α_1_-M as well as a selective ligand of megalin, RBP. Increased urinary excretion of the listed proteins is indicated with an x and with (x) if only trace amounts were observed. No + x indicates that not all the affected patients with this mutation showed similar increased urinary excretion.

^A^Previously described as *c.208-2A>G, skipping of exon 4, fs*[5]. ^B^Previously described by [4].

## Discussion

In the present study, we identified two novel mutations of the *AMN* gene and one novel mutation of the *CUBN* gene as well as three previously described disease-causing mutations through genetic screening of IGS patients from six families. In addition, we performed a detailed analysis of the urinary protein excretion in these patients and investigated effects on receptor function through *in silico* and/or *in vitro* analyses. Table 2 summarises the functional characterisations of the detected mutations.

It was previously proposed that downstream putative transcription start sites of the *AMN* gene were responsible for the non-lethal phenotype of IGS patients with *AMN* mutations in the 5′ region [5] in contrast to the embryonic lethality observed in *Amn-*deficient mice [11]. However, this does not appear to be the case as subsequent reports have identified mutations farther downstream in the *AMN* gene [34]. Instead, it may represent essential differences in embryonic development of humans and mice [44,45]. Human studies of IGS are therefore crucial for understanding the underlying molecular pathology of the clinical manifestations in these patients. This is, however, very difficult due to lack of accessible cubilin- and amnionless-expressing tissues.

In families 1 and 2 we identified the previously described c.208-2A>G mutation of the *AMN* gene. Both families originate from the Mediterranean region (Table 1) where this mutation was previously reported a number of times [5,36-38]. Identification of this mutation in additional families hereby provides further evidence of a founder effect originating from this region [34,46]. The 70 bp intronic deletion of the *AMN* gene identified in family 3 is to our knowledge the largest deletion reported in an IGS patient so far and most likely results in functionally null *AMN* alleles. Compound heterozygous mutations of the *AMN* gene have not been frequently reported [24,35,47,48] but here we report an additional case of a compound heterozygous mutation of the *AMN* gene. In family 4, c.208-2A>G was identified on the maternal allele whereas a novel deletion-insertion of exon 10 (c.1041_1042delinsCTC) was identified on the paternal allele. To our knowledge, this is also the first report of an insertion-deletion mutation in an IGS patient, thus adding to the heterogeneity of the syndrome. Similar to the 70 bp deletion identified in family 3, the deletion-insertion most likely results in a functional null *AMN* allele. Both the previously described Mediterranean founder mutation and the novel *AMN* mutations most likely affect cell surface expression of cubilin (Table 2), as posttranslational modifications and apical membrane expression of cubilin is highly dependent on proper amnionless function and localisation [17,18,20,28].

Genetic screening of family 5 and 6 revealed two distinct missense mutations of the *CUBN* gene. Family 6 is of Finnish origin and here, the previously described FM1 mutation was identified in the affected child. In family 5, however, a novel missense mutation of *CUBN* exon 24 (c.3335G>A, p. G1112E) was identified. The affected residue is located in CUB domain 6 which is part of the IF-B_12_ binding site of cubilin [15,16]. Functional analyses of the G1112E mutation showed intracellular retention of the mutant protein in transfected CHO-K1 cells, most likely caused by detrimental effects on structural integrity of the CUB domain interactions (Table 2). Despite the seemingly similar nature of the two *CUBN* mutations, the functional analyses predict highly distinctive effects on receptor function. This prediction is consistent with the observed differences in urinary protein excretion in the affected patients from the two families (Table 3). In line with previous observations, the patient of Finnish origin (FM1, P1297L) did not show a clear increase in urinary excretion of cubilin ligands (Table 3) [40]. Although previous *in vitro* studies of P1297L suggest that the mutation does not affect the structural integrity of the IF-B_12_ binding site of cubilin [39], limited amounts of cubilin ligands were detected in the urine of this patient. As the interaction sites of very few cubilin ligands have been mapped it is likely that these may have overlapping interaction sites with IF-B_12_.Thus, this could possibly result in decreased affinity for more cubilin ligands besides IF-B_12_ and consequently also in a slightly less efficient proximal tubular function of cubilin.

In contrast, increased urinary excretion of the cubilin ligands; albumin, transferrin, VDBP, apo A-I and α_1_-M was detected in the patients of family 5. This is in line with observations from the patient identified with the IVS-intraCUB6 C>G, FM2 *CUBN* mutation [30-32,40]. Consistent with predictions of disrupted cubilin membrane expression, increased urinary excretion of cubilin ligands was also observed in the patients with *AMN* mutations (Table 3).

Thus, the observations presented here provide additional evidence supporting a correlation between the nature of the IGS-causing mutation and the presence of low-molecular-weight proteinuria. Combined with previous reports of low-molecular-weight proteinuria in IGS patients [24,35-38,40] this constitutes a solid basis for classifying identified IGS causing mutations as either; 1) mutations affecting only receptor recognition of IF-B_12_ in the small intestine or 2) mutations of *CUBN* or *AMN* affecting the overall expression of cubilin on the cell surface resulting in both intestinal IF-B_12_ malabsorption and decreased proximal tubular reabsorption of cubilin ligands from the glomerular ultrafiltrate.

Interestingly, Tanner and co-workers did not identify any IGS-causing mutations beyond *CUBN* exon 28 (corresponding to cubilin CUB domain 8) in a recent large genetic study of families with inherited malabsorption of cobalamin [48]. Furthermore, a recent report described a novel single base pair deletion in *CUBN* exon 53 (c.8355delA; p.S2785fsX19, corresponding to cubilin CUB domain 20) causing only albuminuria but not megaloblastic anaemia [49]. The functional consequences of this particular exon 53 single base pair deletion were not investigated but a similar mutation was recently reported in a group of border collies affected with IGS [50]. Detailed investigations of the cubilin expression and function in these dogs identified reduced expression of cubilin and no evidence of a stable truncated cubilin protein. The vitamin B_12_ status of the patients harbouring the c.8355delA mutation was not analysed in detail but based on the functional investigations in the dog model, it is likely that the patients are deficient in vitamin B_12_ despite the absence of megaloblastic anaemia. Both patients are under 5 years of age and late onset IGS has been reported multiple times in the literature [3]. Because CUB domains 22–27 bind megalin *in vitro*[51] and numerous studies have established that megalin is essential for the endocytic function of cubilin in the proximal tubules [52], one might still speculate, however, that *CUBN* variations in this particular region, resulting in a stable cubilin protein, could constitute a third group of *CUBN* variations that affects only proximal tubular function of cubilin without affecting the intestinal function. Thus, it may be possible that certain *CUBN* mutations may lead to a cubilin related proteinuria without causing IGS. However, based on the current available data, this remains purely speculative and clearly, additional research is needed to further elucidate this.

## Conclusions

In conclusion, the data presented here provide novel insight into the molecular mechanisms underlying the pathology of intestinal IF-B_12_ malabsorption and low-molecular-weight proteinuria of IGS. They furthermore provide additional evidence for a correlation between the nature of the individual disease-causing mutation and the presence of low-molecular-weight proteinuria in IGS patients.

## Abbreviations

α1-M: α1-Microglobulin; apo A-I: Apolipoprotein A-I; ARH: Autosomal recessive hypercholesterolemia; CUB: Complement subcomponents C1r/s, Uegf, and Bmp 1; Dab2: Disabled-2; IF-B12: Intrinsic factor-vitamin B_12_; IGS: Imerslund-Gräsbeck syndrome; MGA1: Megaloblastic anaemia 1; OMIM: Online mendelian inheritance in man; RBP: Retinol-binding protein; SLIM: Site-directed, ligase-independent mutagenesis; VDBP: Vitamin D-binding protein.

## Competing interests

The authors declare that they have no competing interests.

## Authors’ contributions

TS was involved in the study design and interpretation of data. TS also performed genetic screening, *in silico* and *in vitro* molecular analyses, urinary analyses as well as drafting the manuscript. CZ was involved in genetic screening and bioinformatic interpretation of genetic data. OC was involved in the study design and interpretation of data. SA was involved in genetic screening and urinary analyses. MM was involved in the design and interpretation of molecular analyses of identified mutations. IMJ conducted collection of urine samples from healthy control subjects. HK provided 3D illustrations of crystal structures for interpretation of missense mutations and participated in interpretation of data. J-FB was involved in recruitment and clinical investigation of patients. SP was involved in recruitment and clinical investigation of patients. SL was involved in genetic screening. J-MH was involved in genetic screening and bioinformatic interpretation of genetic data. FE was involved in recruitment and clinical investigation of patients. RN, PV, EIC were involved in study design and interpretation of data as well as finalising the manuscript. RK was involved in study design and interpretation of data as well as finalising the manuscript. All authors read and approved the final version of the manuscript.

## Pre-publication history

The pre-publication history for this paper can be accessed here:

http://www.biomedcentral.com/1471-2350/14/111/prepub

## Supplementary Material

Additional file 1 Clinical data on investigated patients. Click here for file
