# Supplementary material for: Detailed investigations of proximal tubular function in Imerslund-Gräsbeck syndrome
Source: BMC Med Genet. 2013 Oct 24;14:111. doi: 10.1186/1471-2350-14-111 (PMC3826550; doi:10.1186/1471-2350-14-111)
Supplement: Additional file 1 — Clinical data on investigated patients. [file 1471-2350-14-111-S1.pdf]

SUPPLEMENTAL DATA

Detailed investigations of proximal tubular function in Imerslund-Gräsbeck Syndrome

CLINICAL DATA ON INVESTIGATED PATIENTS

Abbreviations: MCV (mean corpuscular volume); P_{vitB12} (plasma vitamin B_{12})

Family 1 (patient A):

Is a 6-year-old male, diagnosed at 18 months with anaemia and diarrhoea,
P_{vitB12}: 49 pg/l, folic acid: normal
Anaemia: MCV 97 μm^3
Kidney: proteinuria of 2.16 g/l including microalbuminuria of 1040 mg/l, no homocysteine or amino acids was detected in the urine.
Treatment: vitamin B_{12} injections

Family 2 (patient A):

Is a 16-year-old female
P_{vitB12}: < 100 pg/l, folic acid: normal
Anaemia: MCV 87 μm^3
Kidney: proteinuria of 1. 23 g/l including microalbuminuria of 0.934 mg/l, no homocysteine or amino acids was detected in the urine.
Treatment: vitamin B_{12} injections

Family 2 (patient B):

Is a 27-year-old male.

Family 3 (patient A):

Is a 6-year-old female diagnosed with anaemia.
P_{vitB12} < 100 pg/l, folic acid: normal
Kidney: proteinuria 1.45g/l, microalbuminuria; 900 mg/l, no homocysteine or amino acids was detected in the urine.
Treatment: vitamin B_{12} injections

Family 4 (patient A and B):

Two 6-year-old, female, non-identical twins diagnosed when they were 2 years old with megaloblastic anaemia and low-molecular-weight proteinuria.

P_{vitB12}: < 50 pg/ml for both patients.

Treatment: They are treated weekly per os with 10 µg cyanocobalamin and are currently asymptomatic with normal growth. Kidney function was normal at age 5 years (serum creatinine: 0.41 mg/dl and 0.36 mg/dl; eGFR (ml/min/1.73m^2): 141 and 159) but low-molecular weight proteinuria persists.

Family 5 (patient A):

Is a 10-year-old male diagnosed at age 3 years with anaemia and diarrhoea.
P_{vitB12}: < 60 pg/l, folate: normal
Anaemia: MCV 108 µm^3
Kidney: proteinuria 3.14 g/l and low homocysteine in the urine but normal serum creatinine of 0.3 mg/dl.
Treatment: vitamin B_{12} injections and vitamin D supplementation.

Family 5 (patient B):

Is a 6-year-old male diagnosed at age 18 months with anaemia, urinary infections, and kyphosis of the lumbar region.

P_{vitB12}: < 60 pg/l, folic acid: normal
Anaemia: MCV 80 µm^3
Kidney: low proteinuria and normal serum creatinine of 0.3 mg/dl.
Treatment: vitamin B_{12} injections and vitamin D supplementation.

Family 6 (patient A):
Is a 4-year-old female diagnosed with retarded growth at the age of 3 years with low P_{vitB12} as well as low plasma folic acid.
P_{vitB12}: 21 pg/ml
Folic acid: 60.9 ng/ml
Anaemia: MCV 105 µm^3, haemoglobin 5.4 g/dl.
Kidney: no proteinuria
Treatment: vitamin B_{12} injections every 3 months Cohemin®. Initially, folic acid supplements was also administered but is no longer needed.
